# Association between frequency of dairy product consumption and hypertension: a cross-sectional study in Zhejiang Province, China

**DOI:** 10.1186/s12986-022-00703-2

**Published:** 2022-09-30

**Authors:** Hao Wang, Lingli Chen, Yuan Cao, Kaixu Xie, Chunmei Wang, Pei Pei, Yu Guo, Fiona Bragg, Min Yu, Zhengming Chen, Liming Li

**Affiliations:** 1 Department of NCDs Control and Prevention, Zhejiang Provincial Center for Disease Control and Prevention, #3399 Binsheng road, Binjiang District, Hangzhou, Zhejiang Province China; 2 Department of NCDs Control and Prevention, Tongxiang City Center for Disease Control and Prevention, Tongxiang, China; 3 Peking University Center for Public Health and Epidemic Preparedness and Response, Beijing, China; 4 National Center for Cardiovascular Diseases, Fuwai Hospital, Chinese Academy of Medical Sciences, Beijing, China; 5 Medical Research Council Population Health Research Unit, Nuffield Department of Population Health, University of Oxford, Oxford, UK; 6 Clinical Trial Service Unit and Epidemiological Studies Unit (CTSU), Nuffield Department of Population Health, University of Oxford, Oxford, UK; 7 Department of Epidemiology and Biostatistics, School of Public Health, Peking University Health Science Center, Beijing, China

**Keywords:** Hypertension, Dairy products, Cross-sectional study

## Abstract

**Background:**

Hypertension, a well-known risk factor, contributes to millions of deaths from cardiovascular and renal diseases worldwide. However, evidence on the association between frequency of dairy product consumption and hypertension is inconsistent.

**Methods:**

The data for the present study are from the Tongxiang baseline dataset of the China Kadoorie Biobank prospective study. A total of 53,916 participants aged 30–79 years were included in the final analysis. Multivariable logistic regression was utilized to evaluate the association of dairy product consumption with hypertension, and multiple linear regression was conducted to assess the association of dairy product consumption with systolic and diastolic blood pressure.

**Results:**

Of the 53,916 participants, 2.6% reported consuming dairy products weekly, and 44.4% had prevalent hypertension. After adjusting for socio-demographic status, lifestyle factors, BMI, waist circumference, sleep duration and snoring, when compared with participants who never consumed dairy products, the odds ratios (95% CI) for hypertension among those consuming dairy products less than once per week, and ≥ 1 time per week were 0.85 (0.77–0.95) and 0.74 (0.65–0.84), respectively. The corresponding odds ratios (95% CI) for men were 0.85 (0.71–1.02) and 0.75 (0.61–0.92), respectively (*P*_*trend*_ = 0.001), and for women were 0.88 (0.76–1.01) and 0.77 (0.65–0.91), respectively. (*P*_*trend*_ < 0.001).

**Conclusions:**

In this large epidemiological study, higher frequency of dairy product consumption is associated with significantly lower odds of hypertension among Chinese adults.

**Supplementary Information:**

The online version contains supplementary material available at 10.1186/s12986-022-00703-2.

## Background

Hypertension, along with pre-hypertension and other hazardously high levels of blood pressure, account for 8.5 million deaths from cardiovascular and renal diseases worldwide [1, 2]. Despite a stable global age-standardized prevalence of hypertension, the number of 30–79-year-old adults with hypertension increased substantially from 648 million in 1990 to 1278 million in 2019 [3]. Extrapolation of data from a representative survey of 451,755 adults aged 18 years or older estimated that 245 million Chinese adults had hypertension, and 435 million had pre-hypertension [4]. Furthermore, global hypertension treatment and control rates among women were 47% and 23%, respectively. The corresponding figures for China were 45% and 18%, respectively. Global treatment and control rates of hypertension among men were 38% and 18%, respectively, and in China were 35% and 14% [3]. Hypertension treatment and control rates among Chinese populations were thus lower than global average levels, and prevention and control of hypertension remains a public health priority in China.

Dairy products, abundant in nutrients such as protein, calcium, and vitamins [5], are among the most common foods consumed globally. Although average daily consumption of dairy products among Chinese residents increased from 14.9 g in 1990 to 24.7 g in 2012, consumption levels remain low in comparison with developed countries [6], and China accounts for only 3.5% of world dairy production [7].

Dairy products represent a heterogeneous food group of solid, semi-solid and liquid, fermented or non-fermented foods, each differing in nutrients such as fat and sodium [8]. Furthermore, patterns of dairy product consumption vary greatly across countries and regions. For instance, while people in European Union countries consumed an average of 266 g of dairy products daily [9], in 2020 people in China consumed just 36 g daily [10]. In addition, about 6% of the population consumed yogurt on a daily basis in the United States, while the majority of the population in France consumed at least 1 serving per day [9].

Over past decades, the effects of dairy product consumption on chronic non-communicable diseases and other conditions, including cardiovascular disease, metabolic disorders, diabetes and cancers have been studied [11–15]. While some studies identified dairy product consumption as having favorable effects on health [11, 16], other studies indicated adverse effects [14, 15].

Previous studies examining the association between dairy product consumption and hypertension provided inconsistent results. Several observational studies indicated that dairy product consumption was inversely associated with systolic blood pressure and the risk of hypertension [17–19]. However, null associations were found in other studies [20–22]. Hence, the aim of this study was to examine the association of frequency of dairy product consumption with hypertension using data from the China Kadoorie Biobank (CKB) study in Tongxiang, Zhejiang.

## Methods

### Study population and design

Detailed information about the CKB study design, survey methods and participant characteristics has been reported previously [23–25]. The data utilized in the present study were derived from Tongxiang, one of the 10 regions (5 urban regions and 5 rural regions) included in the CKB study. Between August 2004 and January 2008, 57,704 permanent residents aged 30–79 years were recruited and participated in the baseline survey. The survey comprised a face-to-face interview using a structured questionnaire, physical measurements and blood sample collection. All survey operations were conducted by trained staff using standardized procedures. Individuals with a history of doctor-diagnosed cancer (n = 163), stroke (n = 349), heart disease (n = 464), or diabetes (n = 1380), or with newly diagnosed diabetes at baseline (n = 1432) were not included in the present study. Following these exclusions, 53,916 (22,573 men, 31,343 women) participants remained in the analyses.

### Measurement of outcome variable

All participants were required to avoid eating, drinking alcohol, smoking, and exercising for at least 5 min before taking measurements. Blood pressure was measured on the unclothed right upper arm, in a seated position at least twice using an Omron UA-779 digital sphygmomanometer. Two measurements were undertaken with a 5-min interval between measurements. If the first and second systolic blood pressure (SBP) measurements differed by > 10 mmHg, a third measurement was conducted and the last two measurements recorded. The average of the last two readings was utilized for analyses [26].

Prevalent hypertensive individuals were defined as those with at least one of the following: (1) measured SBP ≥ 140 mmHg, and/or measured diastolic blood pressure (DBP) ≥ 90 mmHg; (2) previous doctor-diagnosed hypertension; or (3) use of antihypertensive medication [27].

### Measurement of exposure variable

Frequency of dairy product consumption was assessed through the question “During the past 12 months, about how often did you eat dairy products (milk, yogurt)?”. Answer options included: “Daily”, “4–6 days/week”, “1–3 days/week”, “Monthly”, and “Never/rarely” (i.e., non-consumers). In analyses, those who chose “Daily”, “4–6 days/week” or “1–3 days/week” were combined into one group (i.e., weekly).

A study comparing the CKB dietary questionnaire with a 12-day 24-h dietary recall (the gold standard) among 432 CKB participants estimated adjusted Spearman coefficients and weighted kappa coefficients for dairy product consumption of 0.47 and 0.75, respectively. The reproducibility of dairy product consumption was tested twice, and the corresponding figures were 0.39 and 0.82, respectively [28].

### Measurement of covariates

A laptop-based questionnaire collected data on socio-demographic characteristics (age, gender, education attainment, marital status, and household income), behavioral lifestyle factors (smoking status, drinking status, physical activity, consumption of fresh fruit and meat, sleep duration and snoring, etc.), personal and family medical history, and menopause status in women.

Participants were categorized into four groups based on their smoking (and alcohol drinking) behaviors: non-smokers (or non-drinkers), former smokers (or former drinkers), occasional smokers (or occasional drinkers), and current smokers (or current drinkers) [29, 30]. Total physical activity was converted into metabolic equivalent of task hours per day (MET-hours/day) on the basis of transport, occupation, housework, and non-sedentary recreation as described previously [31, 32].

Physical measurements were conducted using calibrated instruments by qualified health workers. Standing height was measured to the nearest 0.1 cm with the participant standing erect in bare feet. Weight was measured to the nearest 0.1 kg using the TBF-300 body composition analyzer (Tanita Inc, Tokyo, Japan). Body mass index (BMI) was calculated as weight in kilograms divided by the square of standing height in meters, and obesity was defined as BMI ≥ 25.0 kg/m^2^ [33]. WC was measured to the nearest 0.1 cm with a soft non-stretchable tape measure at the midpoint between the lowest rib and the iliac crest. Abdominal obesity was defined as WC ≥ 85 cm for men, and ≥ 80 cm for women [34]. A non-fasting venous blood sample was collected. Immediate on-site testing of plasma glucose level was undertaken.

### Statistical analysis

SAS version 9.4 was used for all statistical analyses. To ascertain the association between frequency of dairy product consumption and odds of prevalent hypertension, univariate and multivariable logistic regression analyses were utilized. Participants who never consumed dairy products comprised the reference group. Potential confounding factors, including socio-demographic status and lifestyle factors were adjusted for in different models. In model 1, odds ratios (OR_S_) were adjusted for age (continuous) and sex. Model 2 included additional adjustment for educational attainment (no formal school, primary school, middle school, and high school or above) and income (≤ 19,999 yuan, 20,000–34,999 yuan, ≥ 35,000 yuan). Model 3 included additional adjustment for smoking status (never, occasional, former, and current), drinking status (never, occasional, former, and current), physical activity (continuous), meat and fruit intake (daily and non-daily), BMI (continuous), WC (continuous), snoring (never, occasional, and habitual) and sleep duration (continuous). Multiple linear regression analyses were conducted to evaluate the associations of frequency of dairy product consumption with SBP and DBP. In sensitivity analyses, 6 868 participants with self-reported physician-diagnosed hypertension were excluded from the analyses. Stratified analyses by age (30–49 years, 50–79 years), educational attainment (illiterate, primary or above), household income (< 35,000 yuan, ≥ 35,000 yuan), physical activity (< 30 MET-h/d, ≥ 30 MET-h/d), smoking status (current smokers, non-current smokers), alcohol status (current drinkers, non-current drinkers), meat consumption (daily, non-daily), fruit consumption (daily, non-daily), BMI (< 25 kg/m^2^, ≥ 25 kg/m^2^), WC (normal, excessive), sleep duration (< 7.6 h/d, ≥ 7.6 h/d), or menopause status (post-menopausal, pre-menopausal) were also performed. In addition, since the prevalence of hypertension was high among the current study population, Poisson regression models with robust variance were fitted to assess the associations of frequency of dairy product consumption with hypertension, yielding prevalence ratios (PRs) instead of ORs. Statistical significance was set at *P* = 0.05.

## Results

### Characteristics of participants

Participants were, on average, aged 52.5 (SD 9.9) years and had a mean BMI of 22.9 (SD 3.1) kg/m^2^ and a mean WC of 76.5 (SD 9.1) cm. Around 58% were women, 28% were current smokers, 17% were current drinkers, 15% consumed meat daily, 7% consumed fruit daily, and 24% were habitual snorers. Those with higher frequency of dairy product consumption were more likely to be young, female, well-educated, wealthy, to consume meat and fresh fruit frequently, and to sleep for a longer duration. They were less likely to smoke cigarettes, be physically active, or be habitual snorers. There were no significant differences in BMI (*P* = 0.081), WC (*P* = 0.26) or drinking status (*P* = 0.05) according to frequency of dairy product consumption. Among the 53,916 participants, 44.4% (n = 23,921) had prevalent hypertension, and the separate corresponding figures for men and women were 47.3% (n = 10,685) and 42.2% (n = 13,236), respectively. Overall, 94.1%, 3.3%, and 2.6% of participants consumed dairy products never, monthly, and weekly, respectively (Table 1).

**Table 1:** Baseline characteristics of participants according to frequency of dairy product consumption

Characteristics	Overall (N = 53,916)	Frequency of dairy product consumption			*P*_*trend*_
		Never	< 1 Time/week	Weekly	
		(N = 50,746)	(N = 1757)	(N = 1413)	
Mean age (years)^a^	52.5 ± 9.9	52.7 ± 9.8	49.3 ± 10.1	49.7 ± 10.4	< 0.001
Women (%)^b^	58.1	57.7	64.2	65.0	< 0.001
High school or above (%)	43.5	3.3	9.8	16.6	< 0.001
Income ≥ 35,000 yuan (%)	37.8	37.2	44.0	45.5	< 0.001
Current smokers (%)	28.0	28.1	27.1	24.0	< 0.001
Current drinkers (%)	17.1	17.2	14.4	16.0	0.05
Physical activity (MET-h/d)	30.6 ± 15.3	31.0 ± 15.2	26.9 ± 15.5	22.7 ± 14.5	< 0.001
Consuming meat daily (%)	15.2	14.6	19.7	28.6	< 0.001
Consuming fruit daily (%)	6.7	5.4	19.1	34.8	< 0.001
Consuming vegetables daily (%)	93.9	93.9	93.1	93.9	0.66
BMI (kg/m^2^)	22.9 ± 3.1	22.9 ± 3.1	23.0 ± 3.0	23.0 ± 2.9	0.081
WC (cm)	76.5 ± 9.1	76.5 ± 9.1	77.3 ± 8.9	76.7 ± 8.7	0.26
Habitual snoring (%)	24.3	24.4	23.7	21.3	< 0.001
Sleep duration (hours)	7.6 ± 1.2	7.6 ± 1.2	7.7 ± 1.1	7.8 ± 1.1	< 0.001
Women, post-menopause (%)^b^	53.9	53.8	54.0	50.4	0.37

Data were adjusted for age and sex unless specified

*MET* metabolic equivalent of task, *BMI* body mass index, *WC* waist circumference

^a^Adjusted for sex

^b^Adjusted for age

### Association of frequency of dairy product consumption with hypertension

After adjustment for age and sex, in comparison with non-consumers, ORs (95% CI) for hypertension among individuals consuming dairy products less than once per week, and ≥ 1 time per week were 0.83 (0.75–0.92), and 0.70 (0.62–0.78), respectively. After further adjustment for other covariates, including education level, household income, cigarette smoking, alcohol drinking, physical activity, meat and fruit intake, BMI, WC, snoring, and sleep duration, ORs (95% CI) for hypertension among individuals consuming dairy products less than once per week, and ≥ 1 time per week were 0.85 (0.77–0.95) and 0.74 (0.65–0.84), respectively. The corresponding ORs (95% CI) for men were 0.85 (0.71–1.02) and 0.75 (0.61–0.92), respectively (*P*_*trend*_ = 0.001), and for women were 0.88 (0.76–1.01), and 0.77 (0.65–0.91), respectively (*P*_*trend*_ < 0.001) (Table 2).

**Table 2:** Unadjusted and adjusted odds ratios for hypertension associated with frequency of dairy product consumption among adults in Zhejiang

Frequency of dairy product consumption	N. participants	Univariate	Multivariable		
			Model 1	Model 2	Model 3
*Total*					
Never	50,746	1.00	1.00	1.00	1.00
< 1 time/week	1757	0.69 (0.62–0.76)	0.83 (0.75–0.92)	0.84 (0.75–0.93)	0.85 (0.77–0.95)
Weekly	1413	0.59 (0.53–0.67)	0.70 (0.62–0.78)	0.71 (0.63–0.80)	0.74 (0.65–0.84)
*P*_*trend*_		< 0.001	< 0.001	< 0.001	< 0.001
*Men*^*a*^					
Never	21,468	1.00	1.00	1.00	1.00
< 1 time/week	620	0.77 (0.65–0.90)	0.91 (0.77–1.08)	0.91 (0.77–1.08)	0.85 (0.71–1.02)
Weekly	485	0.71 (0.59–0.86)	0.78 (0.65–0.95)	0.78 (0.65–0.95)	0.75 (0.61–0.92)
*P*_*trend*_		< 0.001	0.009	0.007	0.001
*Women*^*a*^					
Never	29,278	1.00	1.00	1.00	1.00
< 1 time/week	1137	0.66 (0.58–0.74)	0.79 (0.69–0.90)	0.81 (0.71–0.93)	0.88 (0.76–1.01) ^b^
Weekly	928	0.55 (0.47–0.63)	0.65 (0.56–0.76)	0.70 (0.60–0.82)	0.77 (0.65–0.91) ^b^
*P*_*trend*_		< 0.001	< 0.001	< 0.001	< 0.001

In model 1, odds ratios were adjusted for age (continuous) and sex. Model 2 included additional adjustment for education level (no formal school, primary school, middle school, and high school or above), household income (≤ 19,999 yuan, 20,000–34,999 yuan, ≥ 35,000 yuan). Model 3 included additional adjustment for cigarette consumption (never, occasional, former, and current), alcohol consumption (never, occasional, former, and current), physical activity (continuous), meat consumption (daily and non-daily), fruit consumption (daily and non-daily), BMI (continuous), WC (continuous), snoring (never, occasional, and habitual), sleep duration (continuous)

^a^Without adjustment for sex, ^b^Additional adjustment for menopause status

### Association of frequency of dairy product consumption with SBP and DBP

After adjusting for the potential confounders included in model 3 (age, sex, socio-demographic and lifestyle factors, adiposity, snoring and sleep duration), higher consumption of dairy products was associated with lower SBP. Compared with non-consumers, the adjusted β coefficients (95% CI) for SBP associated with consumption of dairy products < 1 time/week and weekly were − 1.62 (− 2.54, − 0.69), and − 2.63 (− 3.68, − 1.58), respectively (*P*_*trend*_ < 0.001). The corresponding figures for men were − 2.12 (− 3.64, − 0.60), and − 2.25 (− 3.99, − 0.51), respectively (*P*_*trend*_ < 0.001). A similar association was observed among women (*P*_*trend*_ < 0.001) (Table 3). The frequency of dairy product consumption was also inversely associated with DBP. In comparison with non-consumers, the adjusted β coefficients (95% CI) for DBP associated with consumption of dairy products < 1 time/week, and weekly were − 0.98 (− 1.48, − 0.49), and − 1.40 (− 1.96, − 0.84), respectively (*P*_*trend*_ < 0.001) (Table 4).

**Table 3:** Unadjusted and adjusted *β* coefficients for systolic blood pressure associated with frequency of dairy product consumption among adults in Zhejiang

Characteristics	Frequency of dairy product consumption			*P*_*trend*_
	Never	< 1 Time/week	Weekly	
N. participants (Total)	50,746	1757	1413	
SBP, mmHg	135.6 ± 21.3	130.6 ± 19.4	128.8 ± 19.3	
Unadjusted β (95% CI)	Ref	− 4.97 (− 5.98, − 3.96)	− 6.76 (− 7.88, − 5.65)	< 0.001
Model 1 β (95% CI)	Ref	− 2.62 (− 3.58, − 1.66)	− 4.58 (− 5.65, − 3.52)	< 0.001
Model 2 β (95% CI)	Ref	− 2.36 (− 3.32, − 1.40)	− 4.03 (− 5.11, − 2.95)	< 0.001
Model 3 β (95% CI)	Ref	− 1.62 (− 2.54, − 0.69)	− 2.63 (− 3.68, − 1.58)	< 0.001
N. participants (Men)^a^	21 468	620	485	
SBP, mmHg	136.7 ± 20.6	132.7 ± 18.4	132.8 ± 18.9	
Unadjusted β (95% CI)	Ref	− 4.02 (− 5.65, − 2.39)	− 3.95 (− 5.79, − 2.10)	< 0.001
Model 1 β (95% CI)	Ref	− 2.13 (− 3.71, − 0.55)	− 2.67 (− 4.45, − 0.89)	< 0.001
Model 2 β (95% CI)	Ref	− 2.01 (− 3.60, − 0.43)	− 2.47 (− 4.26, − 0.68)	< 0.001
Model 3 β (95% CI)	Ref	− 2.12 (− 3.64, − 0.60)	− 2.25 (− 3.99, − 0.51)	< 0.001
N. participants (Women)^a^	29 278	1137	928	
SBP, mmHg	134.8 ± 21.7	129.5 ± 19.8	126.8 ± 19.2	
Unadjusted β (95% CI)	Ref	− 5.28 (− 6.55, − 4.00)	− 8.00 (− 9.41, − 6.59)	< 0.001
Model 1 β (95% CI)	Ref	− 2.82 (− 4.02, − 1.61)	− 5.40 (− 6.73, − 4.07)	< 0.001
Model 2 β (95% CI)	Ref	− 2.29 (− 3.50, − 1.08)	− 4.24 (− 5.60, − 2.88)	< 0.001
Model 3 β (95% CI)	Ref	− 1.01 (− 2.18, 0.15)^b^	− 2.26 (− 3.59, − 0.93)^b^	< 0.001

In model 1, odds ratios were adjusted for age (continuous) and sex. Model 2 included additional adjustment for education level (no formal school, primary school, middle school and high school or above), household income (≤ 19,999 yuan, 20,000–34,999 yuan, ≥ 35,000 yuan). Model 3 included additional adjustment for cigarette consumption (never, occasional, former, and current), alcohol consumption (never, occasional, former, and current), physical activity (continuous), meat consumption (daily and non-daily), fruit consumption (daily and non-daily), BMI (continuous), WC (continuous), snoring (never, occasional, and habitual snoring), sleep duration (continuous)

^a^Without adjustment for sex, ^b^Additional adjustment for menopause status

**Table 4:** Unadjusted and adjusted *β* coefficients for diastolic blood pressure associated with frequency of dairy product consumption among adults in Zhejiang

Characteristics	Frequency of dairy product consumption			*P*_*trend*_
	Never	< 1 Time/week	Weekly	
N. participants (Total)	50,746	1757	1413	
DBP, mmHg	80.5 ± 10.7	78.9 ± 10.2	78.1 ± 10.3	
Unadjusted β (95% CI)	Ref	− 1.59 (− 2.10, − 1.08)	− 2.39 (− 2.96, − 1.83)	< 0.001
Model 1 β (95% CI)	Ref	− 1.35 (− 1.86, − 0.84)	− 2.14 (− 2.70, − 1.58)	< 0.001
Model 2 β (95% CI)	Ref	− 1.27 (− 1.78, − 0.77)	− 1.95 (− 2.52, − 1.38)	< 0.001
Model 3 β (95% CI)	Ref	− 0.98 (− 1.48, − 0.49)	− 1.40 (− 1.96, − 0.84)	< 0.001
N. participants (Men)^a^	21 468	620	485	
DBP, mmHg	81.8 ± 11.0	80.7 ± 10.6	80.3 ± 11.1	< 0.001
Unadjusted β (95% CI)	Ref	− 1.05 (− 1.92, − 0.17)	− 1.47 (− 2.47, − 0.48)	< 0.001
Model 1 β (95% CI)	Ref	− 1.06(− 1.94, − 0.18)	− 1.48 (− 2.48, − 0.49)	< 0.001
Model 2 β (95% CI)	Ref	− 1.07(− 1.95, − 0.19)	− 1.50 (− 2.49, − 0.50)	< 0.001
Model 3 β (95% CI)	Ref	− 1.16(− 2.00, − 0.31)	− 1.43 (− 2.40, − 0.46)	< 0.001
N. participants (Women)^a^	29 278	1 137	928	
DBP, mmHg	79.6 ± 10.4	77.9 ± 9.9	76.9 ± 9.6	
Unadjusted β (95% CI)	Ref	− 1.65 (− 2.26, − 1.03)	− 2.60 (− 3.28, − 1.92)	< 0.001
Model 1 β (95% CI)	Ref	− 1.50(− 2.12, − 0.89)	− 2.45 (− 3.13, − 1.77)	< 0.001
Model 2 β (95% CI)	Ref	− 1.28(− 1.89, − 0.66)	− 1.97 (− 2.66, − 1.27)	< 0.001
Model 3 β (95% CI)	Ref	− 0.77(− 1.38, − 0.17) ^b^	− 1.23 (− 1.92, − 0.55) ^b^	< 0.001

In model 1, *β* coefficients were adjusted for age (continuous) and sex. Model 2 included additional adjustment for education level (no formal school, primary school, middle school and high school or above), household income (≤ 19,999 yuan, 20,000–34,999 yuan, ≥ 35,000 yuan). Model 3 included additional adjustment for cigarette consumption (never, occasional, former, and current), alcohol consumption (never, occasional, former, and current), physical activity (continuous), meat consumption (daily and non-daily), fruit consumption (daily and non-daily), BMI (continuous), WC (continuous), snoring (never, occasional, and habitual snoring), sleep duration (continuous)

^a^Without adjustment for sex, ^b^Additional adjustment for menopause status

### Sensitivity analyses

In sensitivity analyses, the association was examined separately among 47,048 participants without a self-reported prior diagnosis of hypertension, the adjusted β coefficients (95% CI) for SBP associated with consumption of dairy products < 1 time/week, and weekly were − 2.02 (− 2.96, − 1.07), and − 2.62 (− 3.69, − 1.56), respectively (*P*_*trend*_ < 0.001). The corresponding figures for DBP were − 1.27 (− 1.78, − 0.75), and − 1.25 (− 1.83, − 0.67), respectively (*P*_*trend*_ < 0.001) (Additional file 1: Table S1). Results of robust Poisson regression analyses indicated that, in comparison with non-consumers, individuals consuming dairy products less than once per week and ≥ 1 time per week had adjusted PRs (95% CI) for hypertension of 0.92 (0.89–0.95) and 0.84 (0.79–0.89), respectively (*P*_trend_ < 0.001) (Additional file 1: Table S2).

### Subgroup analyses

The magnitude of the association between frequency of dairy product consumption and hypertension was largely consistent across subgroups defined by age, education level, household income, physical activity, cigarette smoking, meat consumption, fruit consumption, BMI, WC, sleep duration, and menopause status (*P*_*heterogeneity*_ > 0.05) (Additional file 1: Table S3).

## Discussion

In this large cross-sectional study of Chinese adults, higher frequency of dairy product consumption was associated with lower odds of hypertension and lower systolic and diastolic blood pressure.

### Frequency of dairy product consumption

The China Health and Nutrition Survey found that dairy product consumption increased dramatically from 1.45% in 1989 to 16.84% in 2011 among adults aged 18–59 years in China [35]. In the present study, the proportion of participants consuming dairy products weekly was 2.6%, suggesting dairy product consumption is lower in Tongxiang than in Western, developed countries. This may reflect discrepant dietary patterns between Eastern and Western countries, and may be related to lactose intolerance, which is more common among Chinese populations than among European and Australian populations [36]. The proportion of participants consuming dairy products weekly in Tongxiang is also lower than that in Qingdao, one of the other CKB survey regions, where 58.1% of individuals consumed dairy products weekly, and 33.6% consumed dairy products daily. This discrepancy may reflect urban–rural differences in dairy product consumption, consistent with data from the Chinese national statistical yearbook 2021, indicating that urban residents consumed dairy products more than twice as frequently as residents of rural areas [10]. Consistent with a previous study, participants who consumed dairy products more frequently were more likely to be young, female, well-educated, wealthy, and to more frequently consume meat and fresh fruit [37].

### Relationship of frequency of dairy product consumption with hypertension

Existing literature shows mixed findings on the association of dairy product consumption with the risk of hypertension. One prospective cohort of 40,526 French women, with a median follow-up of 12.2 years, indicated that dairy product consumption was not associated with the risk of hypertension [20]. The Prospective Urban and Rural Epidemiological (PURE) study, examined the association of dairy consumption with incident hypertension among 57,547 adults aged 35 and 70 years, with a median follow-up of 9.1 years, and demonstrated that individuals consuming at least 2 servings of dairy products per day had 11% lower risk of hypertension in comparison with non-consumers [38]. The Singapore Chinese Health Study, including 37,124 participants aged 45–74 years, found an inverse association between dairy intake and risk of hypertension over a decade of follow-up in Chinese people with generally low dairy intake [39]. One meta-analysis of 9 prospective cohort studies from Western countries, with 15,367 incident hypertension cases among 57,256 participants, indicated that each 200 g/day increment in total dairy intake was associated with 3% lower risk of hypertension [40]. The magnitude of association of dairy product consumption with hypertension in the present study thus appeared to be stronger than previous studies [38–40]. One possible explanation for this difference was that the data was obtained from Tongxiang, one of the five rural regions included in the CKB study, since a previous study identified that the magnitude of associations of dairy consumption with components of the metabolic syndrome were stronger in regions with low dairy consumption (China, South Asia, Southeast Asia, and Africa) than in regions with high dairy consumption (North America/Europe, Middle East, and South America) [38].

The exact mechanisms through which dairy consumption may be protective against development of hypertension are not well understood. Various components of dairy products, including calcium, potassium, magnesium, and lactotripeptides, may accelerate renal sodium excretion [41], block calcium channels and reduce intracellular calcium concentrations [42], and increase nitric oxide synthesis [43]. These processes may, in turn, decrease vascular resistance [40], reduce angiotensin I-converting enzyme activity, and reduce blood vessel constriction [44].

## Strengths and limitations

The strengths of the present study include the large sample size, use of a validated dietary questionnaire and standardized data collection procedures, and careful adjustment for established and potential risk factors for hypertension. Several limitations could not be avoided. First, the cross-sectional design restricts establishment of the temporal relationship of dairy product consumption with hypertension. Second, the dietary questionnaire failed to collect information on fat content of dairy products consumed, or on specific dairy types consumed (e.g., milk, yogurt, cheese, butter), limiting further exploration of the observed associations. Third, sodium has been proven to be a well-known risk factor for the development of hypertension. In theory, sodium intake should be adjusted for in the current study. However, in practice, the most accurate measurement technique—average sodium excretion from multiple 24-h urine collections—is impractical in large-scale population surveys such as CKB. Fourth, despite adjustment for multiple established and potential risk factors, residual confounding by other unmeasured or unknown factors could not be ruled out.

## Conclusions

In summary, the present study indicates that higher frequency of dairy product consumption is significantly associated with lower odds of hypertension among Chinese adults. These findings have important public health implications and provide evidence in support of current dietary guidelines recommending dairy product consumption.

## Supplementary Information

**Additional file 1. Table S1.** Unadjusted and adjusted β coefficients for SBP and DBP associated with frequency of dairy product consumption among adults without self-reported physician-diagnosed hypertension in Zhejiang. **Table S2.** Unadjusted and adjusted prevalence ratios for hypertension associated with frequency of dairy product consumption among adults in Zhejiang. **Table S3.** Adjusted odds ratios for hypertension associated with consuming dairy products weekly vs. never according to participant characteristics.

## Data Availability

Details of how to access China Kadoorie Biobank data and details of the data release schedule are available from www.ckbiobank.org/site/ Data + Access.

## Abbreviations

CKB: China Kadoorie Biobank
SBP: Systolic blood pressure
DBP: Diastolic blood pressure
MET: Metabolic equivalent tasks
BMI: Body mass index
WC: Waist circumference
OR: Odds ratio
CI: Confidence interval
PR: Prevalence ratio
PURE study: Prospective Urban and Rural Epidemiological Study

## References

[1] Olsen MH, Angell SY, Asma S, Boutouyrie P, Burger D, Chirinos JA, Damasceno A, Delles C, Gimenez-Roqueplo AP, Hering D (2016). A call to action and a lifecourse strategy to address the global burden of raised blood pressure on current and future generations: the Lancet Commission on hypertension. Lancet.

[2] Zhou B, Perel P, Mensah GA, Ezzati M (2021). Global epidemiology, health burden and effective interventions for elevated blood pressure and hypertension. Nat Rev Cardiol.

[3] Collaboration NCDRF (2021). Worldwide trends in hypertension prevalence and progress in treatment and control from 1990 to 2019: a pooled analysis of 1201 population-representative studies with 104 million participants. Lancet.

[4] Wang Z, Chen Z, Zhang L, Wang X, Hao G, Zhang Z, Shao L, Tian Y, Dong Y, Zheng C (2018). Status of hypertension in China: results from the China Hypertension Survey, 2012–2015. Circulation.

[5] Camfield DA, Owen L, Scholey AB, Pipingas A, Stough C (2011). Dairy constituents and neurocognitive health in ageing. Br J Nutr.

[6] He Y, Yang X, Xia J, Zhao L, Yang Y (2016). Consumption of meat and dairy products in China: a review. Proc Nutr Soc.

[7] Wang Y, Li S (2008). Worldwide trends in dairy production and consumption and calcium intake: is promoting consumption of dairy products a sustainable solution for inadequate calcium intake?. Food Nutr Bull.

[8] de Goede J, Soedamah-Muthu SS, Pan A, Gijsbers L, Geleijnse JM (2016). Dairy consumption and risk of stroke: a systematic review and updated dose-response meta-analysis of prospective cohort studies. J Am Heart Assoc.

[9] Fisberg M, Machado R (2015). History of yogurt and current patterns of consumption. Nutr Rev.

[10] National Bureau of Statistics. China Statistical Yearbook 2021. http://www.stats.gov.cn/tjsj/ndsj/2021/indexch.htm.

[11] Dehghan M, Mente A, Rangarajan S, Sheridan P, Mohan V, Iqbal R, Gupta R, Lear S, Wentzel-Viljoen E, Avezum A (2018). Association of dairy intake with cardiovascular disease and mortality in 21 countries from five continents (PURE): a prospective cohort study. Lancet.

[12] Vissers LET, Sluijs I, van der Schouw YT, Forouhi NG, Imamura F, Burgess S, Barricarte A, Boeing H, Bonet C, Chirlaque MD (2019). Dairy product intake and risk of type 2 diabetes in EPIC-InterAct: a Mendelian Randomization Study. Diabetes Care.

[13] Fumeron F, Lamri A, Emery N, Bellili N, Jaziri R, Porchay-Balderelli I, Lantieri O, Balkau B, Marre M, Group DS (2011). Dairy products and the metabolic syndrome in a prospective study, DESIR. J Am Coll Nutr.

[14] McCann SE, Hays J, Baumgart CW, Weiss EH, Yao S, Ambrosone CB (2017). Usual consumption of specific dairy foods is associated with breast cancer in the Roswell Park Cancer Institute Data Bank and BioRepository. Curr Dev Nutr.

[15] Kakkoura MG, Du H, Guo Y, Yu C, Yang L, Pei P, Chen Y, Sansome S, Chan WC, Yang X (2022). Dairy consumption and risks of total and site-specific cancers in Chinese adults: an 11-year prospective study of 0.5 million people. BMC Med.

[16] Malik VS, Sun Q, van Dam RM, Rimm EB, Willett WC, Rosner B, Hu FB (2011). Adolescent dairy product consumption and risk of type 2 diabetes in middle-aged women. Am J Clin Nutr.

[17] Wang H, Fox CS, Troy LM, McKeown NM, Jacques PF (2015). Longitudinal association of dairy consumption with the changes in blood pressure and the risk of incident hypertension: the Framingham Heart Study. Br J Nutr.

[18] Zong G, Sun Q, Yu D, Zhu J, Sun L, Ye X, Li H, Jin Q, Zheng H, Hu FB, Lin X (2014). Dairy consumption, type 2 diabetes, and changes in cardiometabolic traits: a prospective cohort study of middle-aged and older Chinese in Beijing and Shanghai. Diabetes Care.

[19] Livingstone KM, Lovegrove JA, Cockcroft JR, Elwood PC, Pickering JE, Givens DI (2013). Does dairy food intake predict arterial stiffness and blood pressure in men? Evidence from the Caerphilly Prospective Study. Hypertension.

[20] Villaverde P, Lajous M, MacDonald CJ, Fagherazzi G, Boutron-Ruault MC, Bonnet F (2020). Dairy product consumption and hypertension risk in a prospective French cohort of women. Nutr J.

[21] Dauchet L, Kesse-Guyot E, Czernichow S, Bertrais S, Estaquio C, Peneau S, Vergnaud AC, Chat-Yung S, Castetbon K, Deschamps V (2007). Dietary patterns and blood pressure change over 5-y follow-up in the SU VI MAX cohort. Am J Clin Nutr.

[22] Samara A, Herbeth B, Ndiaye NC, Fumeron F, Billod S, Siest G, Visvikis-Siest S (2013). Dairy product consumption, calcium intakes, and metabolic syndrome-related factors over 5 years in the STANISLAS study. Nutrition.

[23] Chen Z, Lee L, Chen J, Collins R, Wu F, Guo Y, Linksted P, Peto R (2005). Cohort profile: the Kadoorie Study of Chronic Disease in China (KSCDC). Int J Epidemiol.

[24] Chen Z, Chen J, Collins R, Guo Y, Peto R, Wu F, Li L (2011). China Kadoorie Biobank collaborative g: China Kadoorie Biobank of 0.5 million people: survey methods, baseline characteristics and long-term follow-up. Int J Epidemiol.

[25] Li LM, Lv J, Guo Y, Collins R, Chen JS, Peto R, Wu F, Chen ZM (2012). China Kadoorie Biobank Collaborative G: [The China Kadoorie Biobank: related methodology and baseline characteristics of the participants]. Zhonghua Liu Xing Bing Xue Za Zhi.

[26] Lacey B, Lewington S, Clarke R, Kong XL, Chen Y, Guo Y, Yang L, Bennett D, Bragg F, Bian Z (2018). Age-specific association between blood pressure and vascular and non-vascular chronic diseases in 0.5 million adults in China: a prospective cohort study. Lancet Glob Health.

[27] Lewington S, Lacey B, Clarke R, Guo Y, Kong XL, Yang L, Chen Y, Bian Z, Chen J, Meng J (2016). The burden of hypertension and associated risk for cardiovascular mortality in China. JAMA Intern Med.

[28] Qin C, Guo Y, Pei P, Du H, Yang L, Chen Y, Shen X, Shi Z, Qi L, Chen J (2022). The relative validity and reproducibility of food frequency questionnaires in the China Kadoorie Biobank Study. Nutrients.

[29] Liu X, Bragg F, Yang L, Kartsonaki C, Guo Y, Du H, Bian Z, Chen Y, Yu C, Lv J (2018). Smoking and smoking cessation in relation to risk of diabetes in Chinese men and women: a 9-year prospective study of 0.5 million people. Lancet Public Health.

[30] Im PK, Millwood IY, Guo Y, Du H, Chen Y, Bian Z, Tan Y, Guo Z, Wu S, Hua Y (2019). Patterns and trends of alcohol consumption in rural and urban areas of China: findings from the China Kadoorie Biobank. BMC Public Health.

[31] Bennett DA, Du H, Bragg F, Guo Y, Wright N, Yang L, Bian Z, Chen Y, Yu C, Wang S (2019). Physical activity, sedentary leisure-time and risk of incident type 2 diabetes: a prospective study of 512,000 Chinese adults. BMJ Open Diabetes Res Care.

[32] Du H, Li L, Whitlock G, Bennett D, Guo Y, Bian Z, Chen J, Sherliker P, Huang Y, Zhang N (2014). Patterns and socio-demographic correlates of domain-specific physical activities and their associations with adiposity in the China Kadoorie Biobank study. BMC Public Health.

[33] Kanazawa M, Yoshiike N, Osaka T, Numba Y, Zimmet P, Inoue S (2005). Criteria and classification of obesity in Japan and Asia-Oceania. World Rev Nutr Diet.

[34] Group of China Obesity Task Force (2004). The guideline for prevention and control of overweight and obesity in Chineses adults. Acta Nutrimenta Sin.

[35] Wang Y, Jia X, Du W, Wang Z, Wang H, Zhang B (2017). Dairy consumption characteristics among Chinese adult residents from 1989 to 2011. Wei Sheng Yan Jiu.

[36] Chen J, Sai XY (2016). Progress on the research of lactose intolerance. Zhonghua Liu Xing Bing Xue Za Zhi.

[37] Song J, Pan C, Li F, Guo Y, Pei P, Tian X, Wang S, Gao R, Pang Z, Chen Z, Li L (2022). Association between dairy consumption and ischemic heart disease among Chinese adults: a prospective study in Qingdao. Nutr Metab (Lond).

[38] Bhavadharini B, Dehghan M, Mente A, Rangarajan S, Sheridan P, Mohan V, Iqbal R, Gupta R, Lear S, Wentzel-Viljoen E (2020). Association of dairy consumption with metabolic syndrome, hypertension and diabetes in 147 812 individuals from 21 countries. BMJ Open Diabetes Res Care.

[39] Talaei M, Pan A, Yuan JM, Koh WP (2017). Dairy food intake is inversely associated with risk of hypertension: the Singapore Chinese Health Study. J Nutr.

[40] Soedamah-Muthu SS, Verberne LD, Ding EL, Engberink MF, Geleijnse JM (2012). Dairy consumption and incidence of hypertension: a dose-response meta-analysis of prospective cohort studies. Hypertension.

[41] Appel LJ, Brands MW, Daniels SR, Karanja N, Elmer PJ, Sacks FM, American Heart A (2006). Dietary approaches to prevent and treat hypertension: a scientific statement from the American Heart Association. Hypertension.

[42] Park KM, Cifelli CJ (2013). Dairy and blood pressure: a fresh look at the evidence. Nutr Rev.

[43] Flack JM, Calhoun D, Schiffrin EL (2018). The new ACC/AHA hypertension guidelines for the prevention, detection, evaluation, and management of high blood pressure in adults. Am J Hypertens.

[44] Boelsma E, Kloek J (2009). Lactotripeptides and antihypertensive effects: a critical review. Br J Nutr.

