# Supplementary material for: Association between frequency of dairy product consumption and hypertension: a cross-sectional study in Zhejiang Province, China
Source: Nutr Metab (Lond). 2022 Sep 30;19:67. doi: 10.1186/s12986-022-00703-2 (PMC9526303; doi:10.1186/s12986-022-00703-2)
Supplement: Supplementary file 1 — Additional file 1. Table S1. Unadjusted and adjusted β coefficients for SBP and DBP associated with frequency of dairy product consumption among adults without self-reported physician-diagnosed hypertension in Zhejiang. Table S2. Unadjusted and adjusted prevalence ratios for hypertension associated with frequency of dairy product consumption among adults in Zhejiang. Table S3. Adjusted odds ratios for hypertension associated with consuming dairy products weekly vs. never according to participant characteristics. [file 12986_2022_703_MOESM1_ESM.doc]

**Table S1 Unadjusted and adjusted *β* coefficients for SBP and DBP associated with frequency of dairy product consumption among adults without** **self-reported physician-diagnosed hypertension** in Zhejiang

| Characteristics | Frequency of dairy products consumption | | | *P _trend_* |
| --- | --- | --- | --- | --- |
|  | Never | < 1 time/week | Weekly |  |
|  | (N=44 299) | (N=1 517) | (N=1 232) |  |
| SBP, mmHg | 133.0±19.9 | 127.5±17.4 | 126.0±17.6 |  |
| Unadjusted β (95%CI) | Ref | -5.47 (-6.48, -4.46) | -6.99 (-8.11, -5.87) | <0.001 |
| Model 1 β (95%CI) | Ref | -3.22 (-4.19, -2.24) | -4.87 (-5.95, -3.79) | <0.001 |
| Model 2 β (95%CI) | Ref | -2.92 (-3.90, -1.94) | -4.27(-5.36, -3.18) | <0.001 |
| Model 3 β (95%CI) | Ref | -2.02 (-2.96, -1.07) | -2.62 (-3.69, -1.56) | <0.001 |
| DBP, mmHg | 79.6±10.4 | 77.6±9.5 | 77.2±10.0 |  |
| Unadjusted β (95%CI) | Ref | -1.95 (-2.48, -1.42) | -2.39 (-2.98, -1.81) | <0.001 |
| Model 1 β (95%CI) | Ref | -1.76 (-2.29, -1.23) | -2.18 (-2.77, -1.60) | <0.001 |
| Model 2 β (95%CI) | Ref | -1.67 (-2.20, -1.14) | -1.98 (-2.56, -1.39) | <0.001 |
| Model 3 β (95%CI) | Ref | -1.27 (-1.78, -0.75) | -1.25 (-1.83, -0.67) | <0.001 |

In model 1, odds ratios were adjusted for age (continuous) and sex. Model 2 included additional adjustment for education level (no formal school, primary school, middle school and high school or above), household income (≤19,999 yuan, 20 000-34 999 yuan, ≥35 000 yuan). Model 3 included additional adjustment for cigarette consumption (never, occasional, former, and current), alcohol consumption (never, occasional, former, and current), physical activity (continuous), meat consumption (daily and non-daily), fruit consumption (daily and non-daily), BMI (continuous), WC (continuous), snoring (never, occasional, and habitual snoring), sleep duration (continuous).

**Table S2 Unadjusted and adjusted prevalence ratios for hypertension associated with frequency of dairy product consumption among** adults in Zhejiang

| Frequency of  dairy product consumption | N．participants | Univariate | Multivariable | | |
| --- | --- | --- | --- | --- | --- |
|  |  |  | Model 1 | Model 2 | Model 3 |
| Total |  |  |  |  |  |
| Never | 50 746 | 1.00 | 1.00 | 1.00 | 1.00 |
| < 1 time/week | 1 757 | 0.84 (0.81-0.87) | 0.90 (0.87-0.92) | 0.90 (0.87-0.93) | 0.92 (0.89-0.95) |
| Weekly | 1 413 | 0.70 (0.66-0.75) | 0.80 (0.75-0.85) | 0.81 (0.76-0.87) | 0.84 (0.79-0.89) |
| *P _trend_* |  | <0.001 | <0.001 | <0.001 | <0.001 |
| Men ^a^ |  |  |  |  |  |
| Never | 21 468 | 1.00 | 1.00 | 1.00 | 1.00 |
| < 1 time/week | 620 | 0.90 (0.85-0.94) | 0.94 (0.89-0.98) | 0.94 (0.89-0.98) | 0.93 (0.85-0.97) |
| Weekly | 485 | 0.80 (0.73-0.89) | 0.88 (0.80-0.96) | 0.88 (0.80-0.96) | 0.86 (0.79-0.95) |
| *P _trend_* |  | 0.005 | 0.005 | 0.006 | 0.002 |
| Women ^a^ |  |  |  |  |  |
| Never | 29 278 | 1.00 | 1.00 | 1.00 | 1.00 |
| < 1 time/week | 1 137 | 0.81 (0.77-0.85) | 0.87 (0.83-0.91) | 0.90 (0.86-0.94) | 0.92 (0.89-0.96) ^b^ |
| Weekly | 928 | 0.66 (0.60-0.72) | 0.76 (0.70-0.82) | 0.80 (0.74-0.87) | 0.85 (0.78-0.93) ^b^ |
| *P _trend_* |  | <0.001 | <0.001 | <0.001 | <0.001 |

In model 1, odds ratios were adjusted for age (continuous) and sex. Model 2 included additional adjustment for education level (no formal school, primary school, middle school, and high school or above), household income (≤19,999 yuan, 20 000-34 999 yuan, ≥35 000 yuan). Model 3 included additional adjustment for cigarette consumption (never, occasional, former, and current), alcohol consumption (never, occasional, former, and current), physical activity (continuous), meat consumption (daily and non-daily), fruit consumption (daily and non-daily), BMI (continuous), WC (continuous), snoring (never, occasional, and habitual), sleep duration (continuous).

a: without adjustment for sex. b: additional adjustment for menopause status.

**Table S3 Adjusted odds ratios for hypertension associated with consuming dairy products weekly vs. never** according to participant characteristics

|  | N. hypertension | OR_s_ (95%CI) | *P _heterogeneity_* |
| --- | --- | --- | --- |
| Age group (years) |  |  | 0.06 |
| 30-49 | 6 726 | 0.66 (0.54-0.79) |  |
| 50-79 | 16 564 | 0.84 (0.70-0.99) |  |
| Education level |  |  | 0.64 |
| No formal education | 11 581 | 0.70 (0.53-0.93) |  |
| Primary or above | 11 709 | 0.75 (0.65-0.87) |  |
| Household income (yuan) |  |  | 0.78 |
| <35 000 | 14 549 | 0.74 (0.62-0.87) |  |
| ≥35 000 | 8 741 | 0.76 (0.63-0.92) |  |
| Physical activity (MET-h/d) |  |  | 0.35 |
| <30 | 12 634 | 0.72 (0.61-0.84) |  |
| ≥30 | 10 656 | 0.82 (0.65-1.02) |  |
| Smoking status |  |  | 0.49 |
| Current smokers | 6 518 | 0.80 (0.61-1.04) |  |
| Current non-smokers | 16 772 | 0.72 (0.62-0.83) |  |
| Alcohol status |  |  | 0.41 |
| Current drinkers | 4 657 | 0.83 (0.60-1.14) |  |
| Current non-drinkers | 18 633 | 0.72 (0.63-0.83) |  |
| Meat consumption |  |  | 0.31 |
| Daily | 3 255 | 0.82 (0.65-1.04) |  |
| Less than daily | 20 035 | 0.72 (0.61-0.83) |  |
| Fruit consumption |  |  | 0.61 |
| Daily | 1 213 | 0.78 (0.62-0.98) |  |
| Less than daily | 22 077 | 0.73 (0.62-0.85) |  |
| BMI (kg/m^2^) |  |  | 0.72 |
| <25 | 15 714 | 0.76 (0.66-0.88) |  |
| ≥25 | 7 576 | 0.72 (0.57-0.92) |  |
| WC (cm) |  |  | 0.78 |
| Normal | 10 469 | 0.73 (0.59-0.89) |  |
| Obese | 12 821 | 0.75 (0.64-0.89) |  |
| Sleep duration (hours/day) |  |  | 0.27 |
| <7.6 | 10 347 | 0.82 (0.66-1.02) |  |
| ≥7.6 | 12 943 | 0.71 (0.60-0.83) |  |
| Menopause status |  |  | 0.86 |
| Post-menopausal | 19 188 | 0.79 (0.63-0.99) |  |
| Pre-menopausal | 4 102 | 0.77 (0.61-0.96) |  |

OR_s_: odds ratios; CI: Confidence Intervals; BMI: Body Mass Index; WC: Waist Circumference

Odds ratios were adjusted for age (continuous) and sex, education level (no formal school, primary school, middle school and high school or above), household income (≤19,999 yuan, 20 000-34 999 yuan, ≥35 000 yuan), cigarette consumption (never, occasional, former, and current), alcohol consumption (never, occasional, former, and current), physical activity (continuous), meat consumption (daily and non-daily), fruit consumption (daily and non-daily), BMI (continuous), WC (continuous), snoring (never, occasional, and habitual), sleep duration (continuous).
